# Abscopal effect in pulmonary carcinoid tumor following ablative stereotactic body radiation therapy: a case report

**DOI:** 10.1186/s13256-020-02512-8

**Published:** 2020-10-04

**Authors:** Samuel A. Kareff, Jonathan W. Lischalk, Rebecca Krochmal, Chul Kim

**Affiliations:** 1 Department of Graduate Medical Education, MedStar Georgetown University Hospital, 3800 Reservoir Road NW, Washington, DC, 20007 USA; 2 Department of Radiation Medicine, MedStar Georgetown University Hospital, 3800 Reservoir Road NW, Washington, DC, 20007 USA; 3 Department of Pulmonology and Critical Care Medicine, MedStar Georgetown University Hospital, 3800 Reservoir Road NW, Washington, DC, 20007 USA; 4 Georgetown Lombardi Comprehensive Cancer Center, MedStar Georgetown University Hospital, 3800 Reservoir Road NW, Washington, DC, 20007 USA

**Keywords:** Abscopal effect, Neuroendocrine tumor, Pulmonary carcinoid

## Abstract

**Background:**

The abscopal effect was described as early as the 1950s, when untreated tumors demonstrated a response after radiation therapy was delivered to an untreated, distant site. The mechanisms underlying this global response to otherwise localized therapy remain unknown, though there is increasing evidence that increased antigen expression following ablative radiotherapy may play a role.

**Case presentation:**

We report a case of a 69-year-old African American woman with a history of metastatic typical pulmonary carcinoid with multiple lung nodules who had a significant decrease in size of an untreated left upper lobe nodule after stereotactic body radiation therapy to an oligoprogressive left lower lobe lesion.

**Conclusions:**

To our knowledge, this report describes the first case of an abscopal effect in a typical pulmonary carcinoid. Further research is needed regarding the mechanisms responsible for this finding and the role of combining radiation therapy and cancer immunotherapy in patients with pulmonary carcinoid tumors.

## Background

The abscopal effect was described as early as the 1950s, when untreated tumors demonstrated a response after radiation therapy was delivered to an untreated, distant site. The mechanisms underlying this global response to otherwise localized therapy remain unknown, though there is increasing evidence that increased antigen expression following ablative radiotherapy may play a critical role [1]. Neuroendocrine tumors (NETs) encompass heterogeneous types of neoplasms, including typical carcinoid, atypical carcinoid, and high-grade carcinomas such as small cell neuroendocrine carcinoma and large cell neuroendocrine carcinoma [2]. Typical carcinoids usually exhibit an indolent course, whereas atypical carcinoids are associated with a more aggressive disease course [2]. We report one case of an abscopal effect in a patient following stereotactic body radiation therapy (SBRT) to a left lower lobe (LLL) carcinoid NET, one of only two known occurrences in the literature, and the first reported in a patient with a typical pulmonary carcinoid. This case report provides insights into potential future therapeutic strategies for NETs, rare entities with overall incidence of carcinoids in the United States estimated at 5.25 cases per 100,000 population, without many established treatment options that have demonstrated efficacy in late-stage clinical trials [3].

## Case presentation

**Fig. 1:**
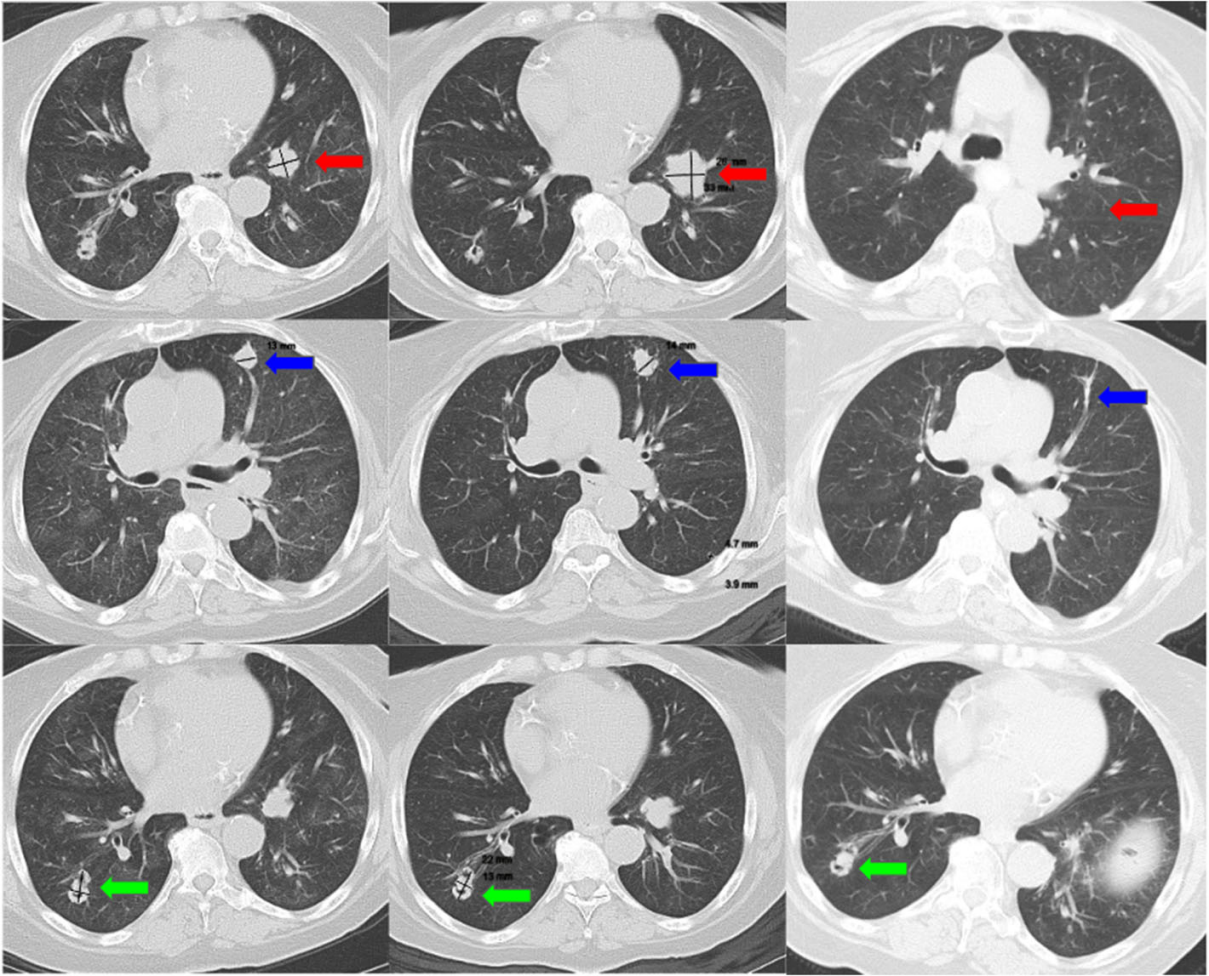
Pulmonary carcinoid lesions throughout the course of therapy (first column = 7 months prior to stereotactic body radiation therapy [SBRT]; second column = 1 month before SBRT, third column = 3 months after SBRT). The patient’s index left lower lobe (LLL; red arrow), left upper lobe (LUL; blue arrow), and right lower lobe (RLL; green arrow) lesions throughout the course of therapy. The first column demonstrates lesions during the time of treatment with lanreotide (7 months prior to SBRT). The second column displays the same lesions 1 month prior to SBRT treatment. The third column demonstrates resolution of the LLL and LUL lesions as well as stable size of the RLL lesion 3 months after SBRT

Our patient was a 69-year-old African American woman with a 72–pack-year smoking history who had originally been diagnosed with a 1.3-cm right lower lobe lung mass about 14 years earlier (clinical stage T1AN0M0 per the eighth edition of the American Joint Committee on Cancer tumor, node, metastasis system). Her symptoms included cough, and the results of her physical examination were largely within normal limits, with the exception of obesity at the time of diagnosis. She underwent a computed tomography (CT)–guided biopsy of the right lower lobe mass, and pathology showed findings consistent with a carcinoid. The results of immunohistochemistry were positive for synaptophysin, Cam5.2, and AE1/AE3. Due to the limitations of the sample, the exact Ki-67 score could not be calculated, but there were rare scattered mitoses and no evidence of necrosis, favoring carcinoid tumor. Over time, she developed bilateral pulmonary nodules that were slowly growing, consistent with metastatic pulmonary typical carcinoid. Her disease had been well controlled with a long-acting formulation of octreotide acetate for 13 years. Surveillance imaging then demonstrated increased disease burden with enlargement of pulmonary nodules and a corresponding increase in chromogranin levels (15 ng/ml in 2015, 227 ng/ml in 2016, 335 ng/ml in 2017, and up to 678 ng/ml in 2019). Everolimus was subsequently initiated but was not tolerated secondary to gastrointestinal side effects despite multiple interruptions and dose reductions. Off-label use of lutetium Lu-177 dotatate was deemed unsuitable, given the patient’s stage IV chronic kidney disease. Everolimus was discontinued 2 months after initiation, and she was transitioned to lanreotide. However, CT performed 3 months after initiation of lanreotide demonstrated a further increase in an index LLL mass (Fig. 1). She underwent video flexible bronchoscopy with endobronchial ultrasound and fiducial placement, which did not yield definitive visualization of an endobronchial lesion. The results of transbronchial needle aspiration, brush biopsies, and transbronchial biopsies were negative for malignancy; however, suspicion remained high for disease progression upon multidisciplinary review.

**Fig. 2:**
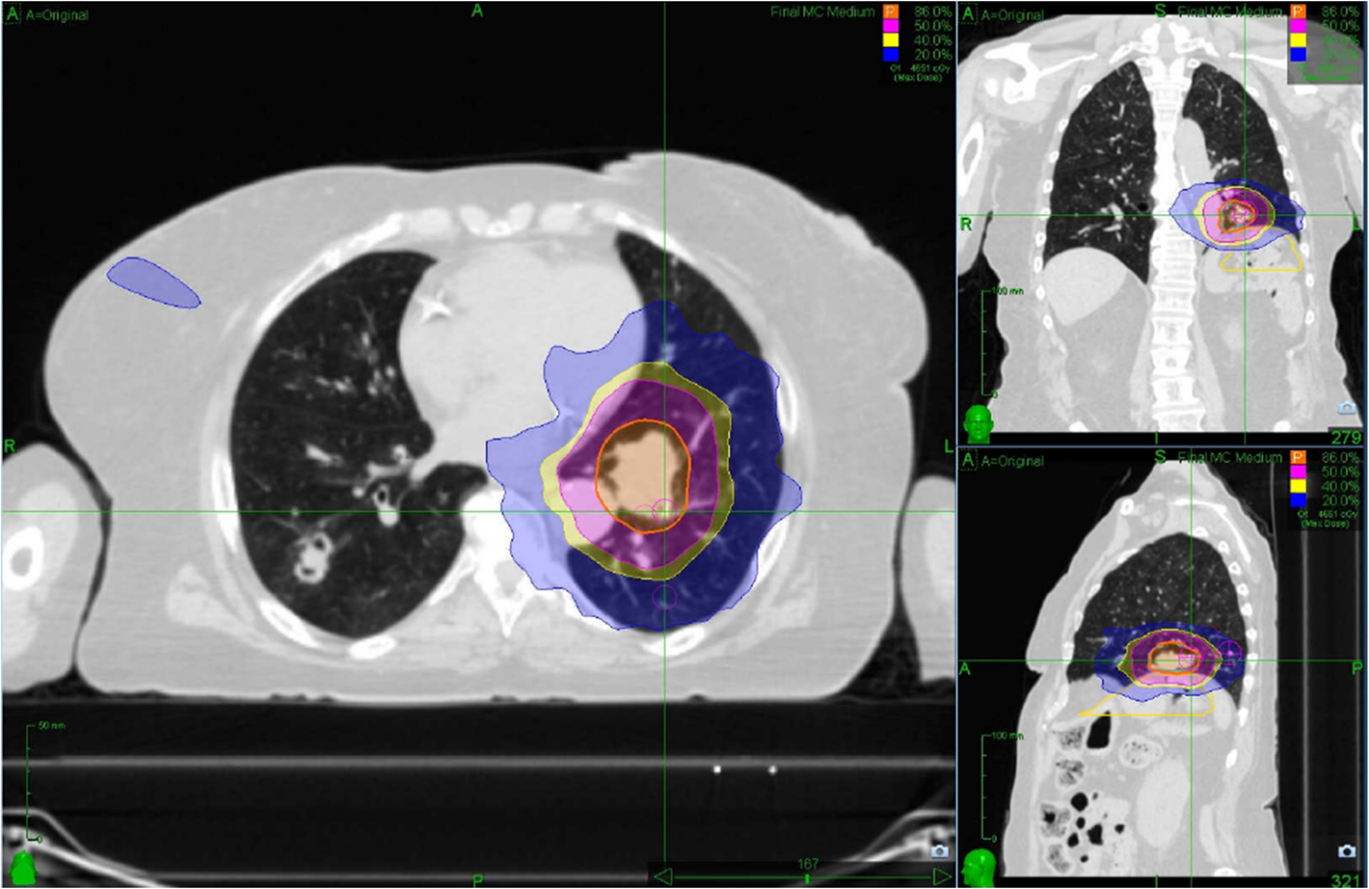
Stereotactic body radiation therapy simulation. Axial, coronal, and sagittal views of the treated left lower lobe carcinoid tumor. Dose color wash superimposed onto the computed tomographic scan demonstrates the prescription dose and range. The blue dose color wash represents the 20% isodose line, which does not overlap with the left upper lobe lesion

Given the anatomical location of the tumor, she was referred to the radiation oncology department, which recommended ablative radiation to a total dose of 4000 cGy in five fractions to the progressing LLL mass with treatments completed every other day to minimize toxicity. Lanreotide was continued during and after the completion of radiation therapy. Repeat imaging 3 months later demonstrated shrinkage of the LLL mass as well as a distant untreated left upper lobe (LUL) nodule. The LUL nodule was largely stable in size prior to radiation (1.4 cm, 1.4 cm, and 1.5 cm measured 10 months, 7 months, and 1 month prior to SBRT, respectively) and shrank to 0.5 cm after SBRT. The location of the LUL lesion was notably anterior and superior to the informal SBRT field used to treat the LLL tumor and received a mean fractional dose of 1.24 Gy, well below the conventional therapeutic dosage (Fig. 2). As such, its regression cannot be ascribed to a classical radiotherapy exposure. She tolerated SBRT without any significant adverse events. Her chromogranin level 3 months after SBRT was 318 ng/ml. After radiation, the patient continued lanreotide without evidence of clinical disease progression. A follow-up imaging study has not been performed yet, owing to her other medical issues and the ongoing coronavirus disease 2019 pandemic.

## Discussion and conclusions

The abscopal effect is currently understood as a distant bystander effect, given the remote nature of non-neighboring cells responding to localized radiotherapy [4]. Although multiple mechanisms have been postulated, current research has focused on various immunologic responses, such as cytokines and chemokines, immunogenic cell death proteins, danger-associated molecular pattern proteins, and even the PD-(L)1 axis [1]. In fact, case series have demonstrated increased reports among known immunogenic tumor types [5]. A PubMed search using the terms “abscopal,” “neuroendocrine,” “carcinoid,” and “bronchial” yielded one case with pulmonary atypical carcinoid [1].

Traditionally, pulmonary NETs and, similarly, less common intrathoracic malignancies have been absent from the literature regarding abscopal effects [6, 7]. Other primary NETs, such as Merkel cell carcinoma and metastatic melanoma, also have been reported to display amenability to the abscopal effect, especially with concurrent immunotherapy [8, 9]. Our patient may have experienced the abscopal effect with increased antigen expression due to SBRT followed by CD8^+^ T cell activation and recruitment in a distant site, resulting in tumor debulking, though the exact mechanism remains unknown [8]. It is intriguing that the abscopal effect was seen in the nearby lesion on the ipsilateral lung with no effect on the contralateral nodes. Shared lymphatic systems in the ipsilateral lung might have played a role, though this proximity of lymphatic systems among lesions exhibiting the abscopal effect is an important factor that needs further investigation. High-dose somatostatin analog treatment has been shown to cause apoptosis in NETs [10]. It is also plausible that lanreotide might have had a role in the abscopal effect seen in this case via generation of tumor antigens through apoptosis. The role of somatostatin analog therapy in boosting the response to cancer immunotherapy warrants further study.

Carcinoid tumors have not responded well to single-agent anti-PD-(L)1 therapy [11, 12]. It has been shown that radiation therapy could potentiate the effectiveness of cancer immunotherapy [13]. Recently, it has been suggested that combining comprehensive irradiation of multiple sites with immune checkpoint inhibitor therapy may represent a more efficacious strategy than single-site irradiation [14]. Given this consideration, combination immune checkpoint inhibitor therapy and radiation therapy such as peptide receptor radionuclide therapy (PRRT) warrants further exploration for treatment of NETs because PRRT is a type of systemic radiotherapy that could treat multiple regions that express somatostatin receptor. A systemic review on the abscopal effect reports a wide range of doses and fractions of radiation therapy in cases of abscopal effect [15]. Finding the optimal dose and fractions of radiation that could potentiate immunotherapy will continue being an important topic, given the widespread use of immune checkpoint inhibitor therapy in the treatment of cancer.

In conclusion, our patient’s case demonstrates the first case of the abscopal effect in typical pulmonary carcinoid. Further research is needed regarding the mechanisms responsible for this finding and the role of combining radiation therapy and immune checkpoint inhibitor therapy in pulmonary carcinoid tumors, especially given the limited approved treatment options in this relatively rare disease [3].

## Data Availability

Not applicable.
